# Mr. Macilwain on Stricture of the Œsophagus

**Published:** 1830-07-01

**Authors:** 


### ON STRICTURE OF THE ŒSOPHAGUS. By Mr. MACILWAIN.

Compared with strictures of the urethra, or even of the rectum, the disease under consideration is very rare. Yet our author affirms that he has seen several cases of this afflicting malady. It usually occurs after the middle period of life. The disease is extremely slow in its progress.

"Stricture of the œsophagus may occur at any part of the tube; but as far as the cases which I have seen, or the preparations which I have examined allow me to offer an opinion, I should say, that they happen most frequently in the upper half of the canal. It is not, perhaps, important, could we do so, to fix the precise point at which they occur most frequently, but it has appeared to me that about opposite the commencement of the trachea is the most frequent situation.

"Examination of the strictured œsophagus presents various degrees of thickening of the tube. Sometimes a mere condensation of it, such as results from chronic inflammation; at others, the diseased part is hard, and when a section is made of it, it appears to be evidently of a carcinomatous structure. In some cases, the thickened portion is very small in extent, and the stricture marked by an acute membranous edge, such as would result from an imperfectly developed valvula connivens, only of a dense structure. I have also seen a fungous growth from the mucous membrane, narrowing its calibre so much as almost to render it impermeable. These appearances may be accompanied by ulceration, and this may occur either on the ORAL, or, more frequently, cardiac side of the stricture, which may be more or less involved in it, or otherwise. The thickening may be an inch or two in extent, and there is one specimen in the museum of the college, in which nearly the whole tube is enormously thickened. A section has been made of it, and the diseased structure is seen interspersed by white fibrous lines, evidently those characterizing carcinomatous deposition. There is one fact which has occurred to me, in reflecting on the various preparations which I have examined, viz. that where the alteration of structure is near the stomach, it is more frequently the consequence of specific disease, than when it is found in any other part of the canal. If this be so, the fact is important, as I shall endeavour to shew, in speaking of the treatment. The remote causes of stricture of the œsophagus are very obscure; in some cases, the first difficulty in swallowing is described by the patient as having succeeded to some catarrhal affection, but more frequently there is nothing to be ascertained very clearly on the subject, neither have I been able, from the cases which I have seen, to connect the disease with any particular temperament."

The causes, however, of stricture of the œsophagus are very obscure. The progress and character of the symptoms are as follow:—

"A patient first finds that occasionally there is difficulty in swallowing, attended with a feeling of spasm, which presently subsides, leaving no inconvenience. For a time this occurs only after considerable periods, which become gradually shorter, but for a long time the patient swallows better some days than others, and frequently without difficulty. At length this becomes so augmented as to induce the patient to masticate his food more particularly, in consequence of fearing that a want of this caution will excite spasm, rather than from any suspicion that there exists already any mechanical obstruction. At this time the spasms usually become easily excited, and more or less distressing, until at length they are so violent as to produce a sensation of suffocation, which alarms and distresses the individual in a most pitiable manner. The face on such occasions being very red, the eyes suffused, and the whole countenance expressive of the utmost anxiety: to these is sometimes added a partial rejection of the food. Experience has by this time most likely convinced the patient that any attempt to swallow firm solids will be unavailing, and the spasm excited by it so certain that the trial is avoided.

"The general agitation of the nervous system is now so considerable, that the very fear as it would seem of spasms renders the individual so excitable that these are sometimes induced without any attempt at deglutition. Mental agitation proceeding from any other cause will excite a paroxysm. In this way, not only at times when it is desirable to take food, but generally, the patient is kept in a greater or less state of agitation and alarm, and there is sometimes considerable pain described as shooting towards the back. This, with the small quantity of food usually taken, is productive of considerable emaciation; the countenance assumes an habitual expression of anxiety, and the whole condition of the patient is truly deplorable. If the case be unrelieved, or if it be of a specific character, the patient sinks under the continued irritation which these circumstances, combined with imperfect nutrition, create and maintain. Towards the latter periods, ulceration takes place, and there is great suffering. The ulceration may be confined to the inner surface of the tube, or may destroy it entirely in the part affected. There is a case in St. Bartholomew's, where the lower end of the œsophagus and a portion of the cardiac orifice of the stomach are thus entirely destroyed."

The treatment of strictured œsophagus must be conducted on the same principles that regulate our conduct in stricture of other mucous canals, with some little modification according to the probability or improbability of the existence of malignant disease. It is of great importance to ascertain this question in all cases, as soon as possible, "since the same diligence which would relieve simple thickening, would, in all probability, aggravate a specific disease, and only hasten its fatal termination." There are, Mr. M. acknowledges, no certain signs by which this knowledge can be acquired; but the probability may be inferred, he thinks, from the following considerations.

"If a patient have suffered for a long time, say, from the earlier periods of life; if the difficulty of deglutition has very slowly and gradually increased, so that it may be fairly accounted for by the ordinary increase of stricture; if there have been no pain, but only distressing spasms, and if an external examination, conducted in a satisfactory manner, discovers no departure from the natural condition of subjacent parts, nor is productive of pain or tenderness; if with this the patient's health be tolerably good, notwithstanding considerable emaciation, and the countenance present no peculiarly unhealthy or disordered tint, but only that expressive of anxiety; if, at the same time, there be no symptom of malignant disease existing in any other part of the body—it may be rationally hoped, that the case is susceptible of relief. If, on the contrary, the first symptoms have occurred late in life; if the progress of the disease has not only been attended with spasms, but with occasional pains when no spasms were present; if the progress have been rapid, not occupying, perhaps, from its commencing symptom, more than a year; or if, having been for a considerable period slow, and unattended by very urgent symptoms, the difficulty of swallowing have somewhat suddenly become augmented, and attended with pain at other times; if with these symptoms the obstruction be near the stomach, and there be occasional rejection of the food, and if the patient be advanced in life — the case is probably one of cancerous disease. When, in addition to all this, there be a sallow countenance, with tenderness in any region of the abdomen, or other evidence of much general or local disorder, the case may (with reference to any practice adopted for its relief) be considered certainly one of specific disease."

The principal objects in the treatment are, to keep the alimentary canal and the œsophagus as tranquil as possible, while the stricture is dilated by the elastic gum or armed bougie according to circumstances. Generally speaking, enemata are more proper than aperients by the mouth in these cases. Leeches and counter-irritation are sometimes useful. The following case concludes the section.

"A lady, æt. 50, of an originally nervous temperament, was brought to me from a considerable distance from the country, for advice, in consequence of difficult deglutition. Her fears were so great on the subject, that she could be by no means persuaded to come to London for advice; this was effected by some friends here giving her an invitation to visit them as the avowed object, the real one being for the purpose I have mentioned. The first time I saw her she was considerably agitated. She gave the following account:—that for many years she had been subject to spasms in her throat, or occasional sensations of impeded deglutition; the precise period was not mentioned, but I have since ascertained it to have been not less than twenty years. These symptoms had increased in frequency and severity until she was unable to swallow any food which was not reduced to a fine pulp, either previous to her taking it, or by careful mastication; and even with these cautions, very small particles would occasionally meet with obstruction, and give rise to very distressing and even alarming spasms, from the feeling of threatening suffocation by which they were accompanied. Mental agitation would sometimes produce them without the aid of any attempt to swallow. With the cautions above mentioned, she was a very long time in taking even a moderate meal, and although naturally thin, she appeared evidently to have suffered further emaciation, partly the result of anxiety, and imperfect nutrition. In addition to all this, society, for obvious reasons, had become irksome to her. Her general state of health appeared in other respects tolerably good, if I except a torpid state of the bowels. The first thing that was done was to evacuate these by small doses of calomel and jalap, with a little ginger, given every four hours until the desired effect was produced. Her choice of food, and the cautions she exercised in taking it, rendered any particular directions on these points unnecessary. On the first attempt at introduction, I could not pass even a small varnished catheter; it seemed to enter the stricture, but would not pass through it. It excited severe spasms, the countenance became suffused, expressive of the greatest anxiety, combined with difficult respiration, the patient appearing in danger of suffocation, and rising from her seat in a very hurried and disturbed manner. Notwithstanding that she could ill manage deglutition of fluids, a mixture composed of powdered valerian, with tinct. castorei and mist. camphoræ, was prescribed, which seemed to quiet the nervous system generally. A tartar-emetic plaster was put along the line of the œsophagus, but this was soon after replaced by a plaister of belladonna, which seemed to be productive of advantage; the stricture was touched with a solution of argenti nitras, conveyed to it by a sponge. Leeches were also applied, and repeated previous to further introduction of the instrument. In about three visits, she could allow the instrument to be pressed lightly on the part for several seconds, and without exciting any such distress or spasm as had at first attended its introduction. At the next visit, I succeeded in passing a moderate-sized catheter, which was increased two sizes. At this period, she proposed leaving town, but said she would stay if I could say the precise time which would be necessary; but as I would not do this, I coincided in her wish of returning home, particularly as I was well acquainted with the ability of the gentleman under whose care she would be. She was extremely anxious to know my opinion of the result of the plan proposed to be pursued. The reasons which determined my answer may be gathered from the foregoing paper; the opinion given was, that by perseverance in the plan she would be ultimately rendered very comfortable. This case is still under treatment, but as far as it has gone it promises to justify the opinion which has been given. As this paper was going to press, I wrote to Mr. Dyer, a very intelligent surgeon, of Ringwood, requesting him to inform me of the progress of the case, and also of the opinion he entertained as to whether the stricture was the result of common thickening or malignant disease. His answer I subjoin:—

"My dear Sir,—As the time is now arrived when you wished to hear from me, I have the gratification of informing you that our patient is still improving: the late changeable weather in some degree affected her general health, so as to excite spasms occasionally, but these I am quite satisfied depended on such circumstances, rather than on the local affection, in which opinion——— now decidedly coincides. I keep to the last sized bougie, and, indeed, adhere altogether to the same plan as when I last wrote, and I do not see that we can deviate from it with any advantage. I often wish you could see her, to witness not only the ease with which the instrument passes,* but particularly the improvement in her general health, occasioned by its use enabling her to take her food in greater quantities, and with so much more comfort. With respect to the point you hint at (which, indeed, is a most important one), whether the obstruction be the result of common thickening consequent on chronic inflammation, or whether it be the effect of any specific disease, I should certainly say (bearing in mind the long duration of the affection, certainly more than twenty years,) this case has arisen from the former cause; for, had it been the latter, surely other symptoms would have arisen long ere this, and if not spontaneously, the means to which we have had recourse, would have been sufficient to have excited them, and thus, instead of having lessened the mischief (which is beyond doubt) to have increased it. I think, therefore, there cannot be any question as to the nature of the disease, or that we have every prospect of ultimate success in rendering our patient comfortable. She is, unfortunately, of a nervous, irritable habit, easily excited, which has increased the difficulty with which we have had to contend."

Mr. M. does not allude to the use of iodine in this affection. We fear that the disease is more frequently increased than improved by the interference of bougies.

* "I sent this instrument to Mr. Dyer. As nearly as I can recollect, it is something less than half an inch in diameter."

